# The effect of musical practice on gesture/sound pairing

**DOI:** 10.3389/fpsyg.2015.00376

**Published:** 2015-04-02

**Authors:** Alice M. Proverbio, Lapo Attardo, Matteo Cozzi, Alberto Zani

**Affiliations:** ^1^NeuroMi - Milan Center for Neuroscience, Department of Psychology, University of Milano-Bicocca, Milan, Italy; ^2^Institute of Bioimaging and Molecular Physiology, National Research Council, Milan, Italy

**Keywords:** music learning, visual feedback, auditory processing, multimodal coding, audiomotor, visuomotor, mirror system

## Abstract

Learning to play a musical instrument is a demanding process requiring years of intense practice. Dramatic changes in brain connectivity, volume, and functionality have been shown in skilled musicians. It is thought that music learning involves the formation of novel audio visuomotor associations, but not much is known about the gradual acquisition of this ability. In the present study, we investigated whether formal music training enhances audiovisual multisensory processing. To this end, pupils at different stages of education were examined based on the hypothesis that the strength of audio/visuomotor associations would be augmented as a function of the number of years of conservatory study (expertise). The study participants were violin and clarinet students of pre-academic and academic levels and of different chronological ages, ages of acquisition, and academic levels. A violinist and a clarinetist each played the same score, and each participant viewed the video corresponding to his or her instrument. Pitch, intensity, rhythm, and sound duration were matched across instruments. In half of the trials, the soundtrack did not match (in pitch) the corresponding musical gestures. Data analysis indicated a correlation between the number of years of formal training (expertise) and the ability to detect an audiomotor incongruence in music performance (relative to the musical instrument practiced), thus suggesting a direct correlation between knowing how to play and perceptual sensitivity.

## Introduction

Research has shown that learning to play a musical instrument has dramatic effects on cognition (Schlaug et al., 2005), even after only a few years of training. The beneficial effects of musical training are not limited to enhancement of musical skills [such as, note coding and staff reading (e.g., Proverbio et al., 2013; Wong et al., 2014)] but extend to many other skills, including finger tapping (Braun Janzen et al., 2014), visual memory (Rodrigues et al., 2014), auditory memory (Cohen et al., 2011), speech in noise perception (Strait and Kraus, 2011), auditory temporal processing (Bishop-Liebler et al., 2014), dexterity of finger movements (Furuya et al., 2014), reading skills (Tierney and Kraus, 2013), gesture imitation (Spilka et al., 2010), and non-verbal reasoning (Forgeard et al., 2008). By increasing the number of years of constant practice (musical expertise), the musical ability is thought to improve monotonically, independent of individual musical talent (which naturally affects achievement). Indeed, according to Ericsson et al. (1993), “many characteristics once believed to reflect innate talent are actually the result of intense musical practice extended for a minimum of 10 years.” Interestingly, Groussard et al. (2014) conducted a regression study on 44 non-musicians and amateur musicians with 0–26 years of musical practice with a variety instruments to identify which brain areas undergo gray matter changes as a function of expertise. They found that some brain areas underwent volume changes after only a few years of musical practice, whereas others (especially auditory and motor areas such as the superior temporal and supplementary motor areas) required longer practice before they exhibited changes, thus suggesting a long-lasting learning process.

It is widely agreed that the length of time spent learning a musical skill is a crucial factor in development of expertise (Hallam, 1995, 1998, 2013). Some researchers have attempted to quantify the amount of time it takes to become an expert in a particular skill (e.g., Sosniak and Perlman, 1990). The current estimate (Ericsson et al., 1993) is approximately 10,000 h, which equates to approximately 10 years of practice, although this can vary as a function of the individual talent and aptitude. A recent study examined which factors best predict musical achievement. A large group of instrumental music students was evaluated by their teachers and through a questionnaire; data analysis showed that the level of expertise attained was best predicted by time spent learning (Hallam, 2013).

Although numerous studies have ascertained the presence of audiomotor representations in skilled musicians (e.g., Baumann et al., 2007), little information is available on the development of such representations during learning. In addition, practice-dependent neural plasticity seems to be very sensitive to the technical characteristics of the instrument played. For example, violinists have a greater cortical representation of the left hand than controls (Elbert et al., 1995), and the amount of cortical reorganization is correlated with the age at which the person began to play. Similarly, expert trumpeters display stronger neural interactions between the auditory and somatosensory input on the lips (Schulz et al., 2003) compared to other instrumentalists. The development of audiovisual associations as a result of musical expertise has been shown by Hasegawa et al. (2004), who investigated how pianists can identify familiar pieces of music by watching the key-touching movements of the hands. Naïve groups with little or no experience of piano playing were compared with trained pianists. While pianists were able to identify the music played in a silent video from hands movements, naïve subjects were not, and this was associated with the activation of the left planum temporalis (PT), supporting the representation of sound. It was hypothesized that piano practice induced the formation of new connections between visuomotor and auditory information in the skilled brain and that the activation of the left PT in pianists may be related to the process of association between the rapid movement of the fingers and the sequences of sounds corresponding to the key pressed. Taken together, these findings show that the study of a musical instrument can lead to obvious phenomena of cortical reorganization in response to the increased need for multisensory processing specific to music performance. While this is a well-known fact, knowledge is scarce about intermediate proficiency levels and whether those associations are formed during the early stage of learning or require a much longer period.

The present study aimed to investigate whether formal music training enhanced multimodal associations (auditory, motor, and visual associations) in violinists and clarinetists, as well as whether this ability was acquired within the first few years or, instead, gradually during a period of 2–18 years of study. To this end, congruent vs. incongruent musical stimuli were presented to pupils at different stages of education (grades) to determine whether the ability to play different musical instruments can modify the way the instrument-specific sensory information is processed. Music conservatory violin and clarinet students of pre-academic and academic levels with different chronological ages, ages of acquisition (AoAs) of musical ability, and academic degrees were tested. They were shown videos in which a violinist or a clarinetist played the same musical score on the instrument played by the viewer. Pitch (Hz), intensity (dB), tempo (beats per minute), and sound duration (ms) were matched across instruments. In one condition, the images and soundtrack were coherent, whereas in another condition, musical gestures were accompanied by a perfectly synchronized soundtrack that did not match in pitch with the sounds heard. This manipulation was intended to stimulate the recognition of an inconsistency in the relationship between sound and gesture, specifically in musicians who have developed motor ability for that specific instrument (therefore the violinists for the violin but not the clarinet, and vice versa).

## Materials and Methods

### Participants

Nineteen musicians participated in this experiment: 10 violinists and 9 clarinetists, all from *Milan Conservatory “Giuseppe Verdi.”* Regardless of their age and grade, violinists tended to have more experience than clarinetists. Participants’ chronological age ranged from 14 to 24 years and averaged 17.68 years (SD = 3.2). The AoA of musical ability varied from 5 to 15 years across musicians and averaged 8.5 years. The years of study of the instrument ranged from 2 to 18 years. The chronological age of musicians averaged 16.8 years (SD = 2.94) for clarinetists and 18.67 years (SD = 3.35) for violinists.

**FIGURE 1:**
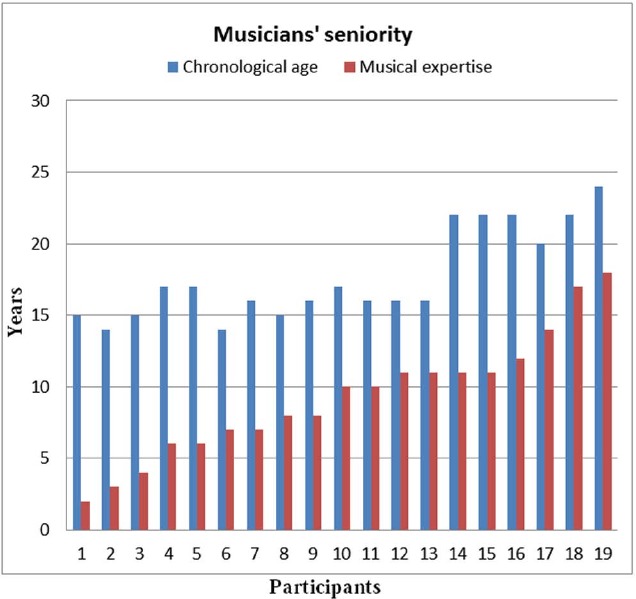
**Study participants with their chronological ages, listed in ascending order as a function of the years of study devoted to their musical instruments**.

All participants had normal visual and auditory skills. Figure 1 shows the relationship between chronological age and the years of study of the instrument for each subject, listed in ascending order. Informed and signed consent was obtained for all participants (for underage students, consent was obtained from their parents). The two musicians who used their personal instruments to perform the musical scores used as stimuli provided release notes for audiovisual material and were moderately compensated. Experiments were conducted with the understanding and written consent of each participant according to the Declaration of Helsinki (BMJ 1991; 302:1194) with approval from the Ethical Committee of University of Milano-Bicocca in compliance with APA ethical standards for the treatment of human volunteers (1992, American Psychological Association).

### Stimuli and Material

**FIGURE 2:**
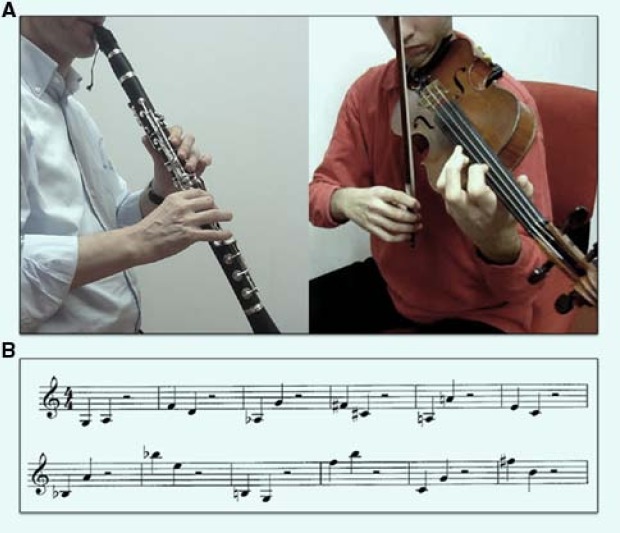
**(A)** Frames taken from the video clips for the clarinet and violin, respectively. The lateral view allowed vision of the tonehole (above the musician’s left thumb) for the clarinet, while the seated position for the violinist allowed a clear view of the keyboard and the right arm in its entire range of extension. **(B)** An excerpt of the musical score played.

A musical score of 200 measures was created (in 4/4 time), featuring 84 single-note measures (1 minim) and 116 double-note measures (2 semiminims). Figure 2B shows an excerpt of the score. Each combination of sounds was also absolutely unprecedented. Six measures were created specifically for the violin, which extends higher than the clarinet, and they were executed one octave lower for the clarinet, which extends lower. Stimulus material was obtained by videotaping a clarinetist and a violinist performing the score. As he played, the actor violinist was filmed in a frontal perspective, so that the movements of the bow and the positions of the fingers on the keyboard were clearly visible. The violinist was seated and slightly farther from the camera than the clarinetist, so that when the left arm was fully extended (in the bow-down position) the image fell within the photographic frame. For the clarinetist, much preliminary testing led to the choice of filming him from a side view (profile). In fact, the clarinet’s tonehole (which is an important clarinet feature placed under the instrument, near the left thumb) remains hidden by the instrument in a frontal view, while it is well visible in lateral view. This is of considerable importance because, when activated, the tonehole raises the note played by one octave and a perfect fifth (see a frame in Figure 2). The position of the musician never changed during recording, which took place in a quiet classroom. Music was executed non-legato, and moderately vibrato on the violin (metronome = BPM 60) for approximately 2 s of sound stimulation for musical beat. The two videos (one for each instrument) were subsequently segmented into 200 movie clips per instrument (for a total of 400 videos). Two clips per instrument were discarded for technical reasons (for a total of four stimuli discarded). Each clip lasted 3 s: during the first second, the musician was getting ready to play (no sound yet), and he played for 2 s thereafter. Therefore, each video clip lasted 3 s, the sound started after 1 s, and it lasted 2 s. The two stimulus classes were equiluminant. Audio sound values were normalized to –16 dB using *Sony Sound Forge 9.0* software, by setting a fixed value of RMS (root mean square of a sound correspond to the perceived intensity recorded at intervals of 50 ms). To obtain an audiovisual incongruence, for half the videos, the original sound was substituted with that from the next measure (which was intentionally created to be sufficiently different) using *Windows Movie Maker 2.6*.

### Procedure

The 396 stimuli were divided into two groups based on the instrument being played, and they were randomly mixed in a PowerPoint presentation. The stimuli were presented to students or musicians found in the clarinet and violin classes at the Conservatory, waiting for their lessons. Participants were comfortably seated in front of a portable notebook PC in an empty testing room and wore headphones for sound stimulation. The task was to evaluate whether the sound-gesture video clip combinations were correct or not by using a 3-point Likert scale (2 = congruent; 1 = I am unsure; 0 = incongruent). Judges evaluated only video clips for the instrument they knew: violinists judged only violin video clips, and clarinetists judged clarinet clips. The aim of the validation test was to ensure that targets (incongruent clips) were easily identifiable by musicians at various levels of expertise, ranging from the pre-academic level through mastery and beyond. The results of this validation were crucial for an EEG study later performed on professional musicians (Proverbio et al., 2014).

### Data Analyses

For each subject, the percentage of errors (incorrect and “I am unsure” responses) was arcsine transformed (for statistical treatment) and underwent a repeated measures analysis of variance of which the factor was Expertise, dependent on the years of study of the instrument (moderate expertise from 2 to 8 years, high expertise from 9 to 18 years).

Furthermore, individual error frequency was correlated with musicians’ chronological age (in years), and with the musical expertise (in years) computed on the basis of the referred AoA of musical ability. The Pearson product-moment correlation coefficient (*r*) was computed for the two classes.

In this study the years of musical expertise, rather than for example “estimated hours of practice” was considered as a very predictive variable in that all participants were actual students (or ex-students) of *Conservatorio di Milano Giuseppe Verdi*, and therefore shared the same teachers, classes organization, and overall study workload per day.

## Results

**FIGURE 3:**
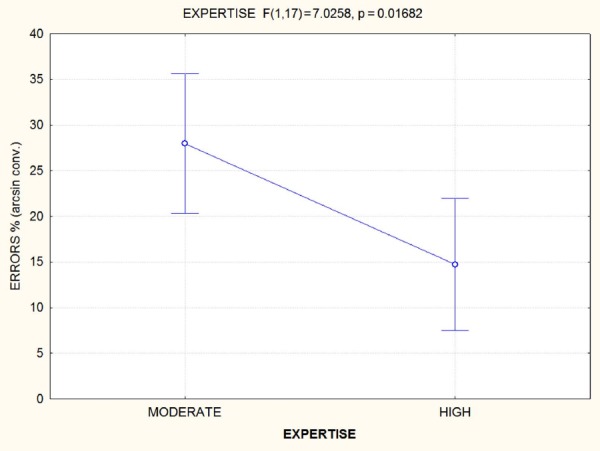
**Accuracy data and statistical significance for *expertise* factor.** After 8 years of training, the ability to detect an audiovisual incongruence in a musical performance underwent a dramatic improvement, thus suggesting an enhancement of multisensory associations as a result of intense practice.

The analysis of variance yielded the significance of Expertise (*F*_1,17_ = 7.03; *p* = 0.0168), indicating a strong effect of the number of years devoted to musical studies on the ability to detect audiovisual incongruences (see Figure 3).

**FIGURE 4:**
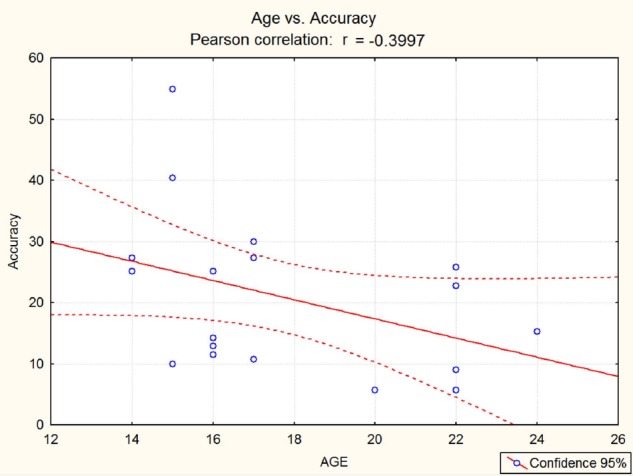
**Pearson’s correlation between musicians’ chronological age (in years) and their accuracy in the audiovisual incongruence detection test y (error frequency %).** An inverse correlation was found (*p* < 0.05) with a small size effect (*r* = –0.4).

**FIGURE 5:**
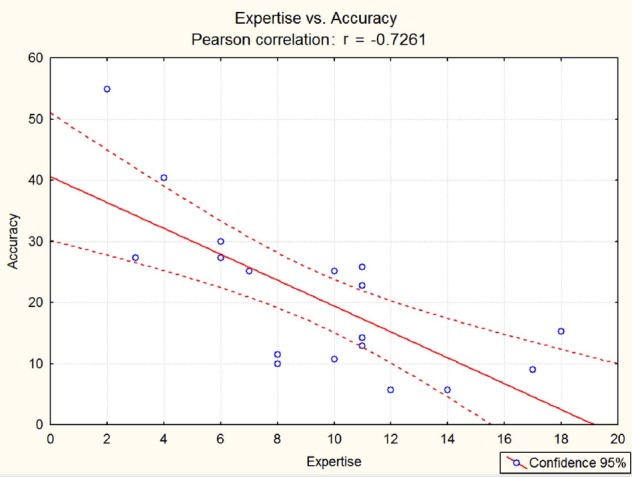
**Pearson’s correlation between years of musical expertise [estimated on the basis of the referred age of acquisition (AOA) of musical ability] and error frequency (%).** An inverse correlation was found (*p* < 0.05) with a large size effect (*r* = –0.73).

The results of Pearson’s correlation was weakly significant in terms of chronological age (*r* = –0.4; *p* < 0.05), as displayed in Figure 4, and strongly significant in terms of years of expertise (*r* = –0.7261), as shown in Figure 5. In both cases, a significant inverse correlation was found between number of years and error frequency (arcsine converted): older or more expert pupils, showed lower error frequencies in the audiovisual incongruence test. It should be remarked that the effect of expertise was much stronger than that of chronological age.

## Discussion

Overall data analysis indicated a strong effect of the number of years devoted to musical studies (expertise factor) in the ability to detect audiovisual incongruences, which is consistent with the available neuroimaging literature showing that long-term musical training alters the basic audiovisual temporal processing in the skilled musician (Paraskevopoulos et al., 2012; Lu et al., 2014; Proverbio et al., 2014). Indeed, to our knowledge, all such studies have been performed comparing the skilled brain of musicians to adult non-musicians [see for example Bermudez and Zatorre (2005) and Barrett et al. (2013), for a review], and no knowledge is available about the degree of musical expertise and the formation of new associations within the learning brain. In a combined psychophysics-fMRI study (Lee and Noppeney, 2011) investigating how sensorimotor experience molded temporal binding of auditory and visual signals, musicians showed increased audiovisual asynchrony responses for music in neural circuitry including the superior temporal sulcus (auditory areas) and the premotor and cerebellar cortex (motor and sensory motor areas). In another fMRI study (Petrini et al., 2011) performed on skilled drummers, the stimulus sound was synchronized or desynchronized with drumming strikes to provide an audiovisual incongruence (as in the present study). The results showed that drummers’ brain activation was reduced in motor and action representation brain regions when the sound matched the observed movements and was similar to that of novices when sound was mismatched, thus indicating that brain functions in action–sound representation areas are modulated by multimodal action expertise. To our knowledge, similar experiments have not been performed in violinists or clarinetists. In our study, the ability to detect a visuomotor incongruence correlated with the numbers of years of study of violin or clarinet. This pattern of results seems to support our initial hypothesis that the acquisition of musical ability gradually leads to the development of visuomotor associations allowing the visual recognition of an auditory incongruence. It is therefore likely that, because of neuroplasticity, a long period of training leads to the development of audio and visuomotor associations able to integrate multimodal information in a very specific manner (e.g., Schulz et al., 2003; Hasegawa et al., 2004; Baumann et al., 2007; Proverbio et al., 2013). Furthermore, the importance of visual information processing during musical execution for coordinating performance has been shown in musicians playing simultaneously (in orchestra, for example, or in quartets, as in the study by Badino et al., 2014).

The present data show not only that multisensory audio visuomotor processing is crucially involved in music learning but that the ability to process music-related multisensory information progresses constantly even after many years of study, as demonstrated by the significant difference in the performance between moderate and highly expert pupils (from 9 to 18 years of practice). This suggests a very long-lasting period of cortical plasticity induced by musical learning and intensive practice. Furthermore, the data suggest an effect of expertise independent of chronological age *per se*, which correlated with performance, but with a smaller effect size (*r*) than expertise. In fact, it has been shown that musicians who began training early show better musical performance and greater changes in auditory and motor regions of the brain than late learners (Watanabe et al., 2007; Penhune, 2011; Bailey and Penhune, 2012). Therefore, training during a sensitive period in development may have greater effects on brain structure and behavior than training later in life. Although this is certainly true, our data indicate that the improvement in performance cannot be ascribed to brain maturation *per se* and that it depends on practice. The strong correlation between years of practice and achievement indicates a crucial role of “deliberate” practice in predicting music achievement (Sosniak and Perlman, 1990; Ericsson et al., 1993; Hallam, 1995, 1998, 2013). According to Ericsson et al. (1993), the amount of time an individual is engaged in “deliberate” practice (which, in the present study, corresponds to the years of formal study at the *Milan Conservatory*) is monotonically related to the quality of that individual’s performance. Therefore, to reach the highest levels of expertise, the amount of practice should be maximized. According to this view, the exercise is at least as important as genetics and predisposition in determining performance (cf. the nurture vs. nature hypothesis) because all individuals show a dramatic benefit from practicing their skill (as in the case of the present study). While behavioral genetic analyses indicate that a certain proportion of individual differences in performance are associated with genetic factors (Mosing et al., 2014; Hambrick and Tucker-Drob, 2015), the instrument-specific effect of musical expertise on perceptual sensitivity to incongruence that is described here, as well as in other studies, hints at a crucial role of musical practice in shaping the musical brain functions and behavior (the nurture hypothesis, Wan and Schlaug, 2010).

Naturally, the effects on neuroplasticity are not visible only in musicians but also in other skilled populations, such as athletes or dancers (Brutsaert and Parra, 2009). For example, Proverbio et al. (2012) investigated the effect of expertise in professional basketball players vs. naïve students and measured how this affected their sensitivity in detecting an incorrect motor action with an N400 paradigm. Similarly to the present study, the skilled population (experts) showed a better sensitivity to perceptual incongruence, presumably through the enhancement of multimodal associations.

In conclusion, this study shows that the ability to detect an audiovisual incongruence in musical performance is an excellence predictor of musical expertise with a given instrument, thus suggesting a direct correlation between knowing how to play and sensitivity in audiovisual perception (musical *cerebral resonance*, Harris and de Jong, 2014), also shown in neuroimaging (Proverbio et al., 2014) and behavioral studies (Petrini et al., 2009; Bishop and Goebl, 2014).

**FIGURE 6:**
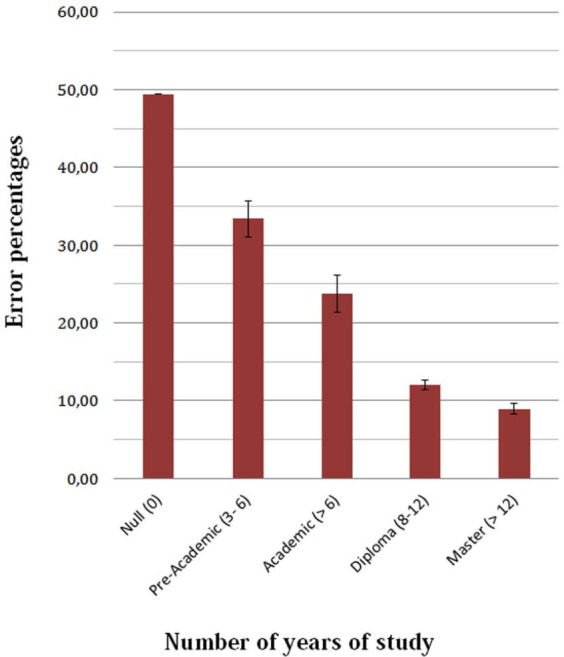
**How sensitivity to audiovisual incongruence progresses as a function of the number of years of musical study and motor practice. Error percentages are plotted along with standard deviations.** The first bar refers to a group of controls (*N* = 4) with null musical education, the last one to a group of teachers and experienced musicians (*N* = 4).

One of the possible limitations of the present study is that students were not tested with the unfamiliar instruments, which would have provided direct clues about the possible instrument-specificity of the effects. However, we did test four control students (non-musicians, but amateur guitarists and singers) and their performance is reported in Figure 6. Their hit percentage (49.4%) was equal to chance, which supports the hypothesis that the ability to perceive an audiovisual incongruence depends on musical practice with the specific musical instrument. In Figure 6, it can also be appreciated the excellent performance of older and more experienced teachers (with master degree), showing that the refinement of perceptual sensitivity continues after many years of study (>12 years) and is a lifelong process. The data of controls and teachers were not statistically analyzed with that of students, because of excessive groups’ heterogeneity.

Direct evidence of the instrument-specificity of this effect has been provided in an ERP study performed on professional musicians with the same stimulus set (Proverbio and Orlandi, 2015). Here, clarinet and violin video clips were presented to professional clarinetists and violinists, as well as controls. Participants were asked to count the number of notes they perceived being played. The results showed a dramatic effect of instrument familiarity on several ERP components, suggesting a direct relation between knowing how to play (motor ability) and perceptual sensitivity for musical gestures.

### Conflict of Interest Statement

The authors declare that the research was conducted in the absence of any commercial or financial relationships that could be construed as a potential conflict of interest.
